# Neonatal Outcome After Preeclampsia and HELLP Syndrome: A Population-Based Cohort Study in Germany

**DOI:** 10.3389/fped.2020.579293

**Published:** 2020-10-12

**Authors:** Verena Bossung, Mats Ingmar Fortmann, Christoph Fusch, Tanja Rausch, Egbert Herting, Isabelle Swoboda, Achim Rody, Christoph Härtel, Wolfgang Göpel, Alexander Humberg

**Affiliations:** ^1^Department of Obstetrics and Gynaecology, University Hospital of Schleswig-Holstein, Lübeck, Germany; ^2^Department of Paediatrics, University Hospital of Schleswig-Holstein, Lübeck, Germany; ^3^Department of Paediatrics, Paracelsus Medical School, General Hospital of Nuremberg, Nuremberg, Germany; ^4^Institute of Medical Biometry and Statistics, University Medical Centre of Schleswig-Holstein, University of Luebeck, Luebeck, Germany

**Keywords:** preterm, VLBWI, preeclampsia, HELLP, intrauterine growth restriction

## Abstract

**Aim:** To analyze short term outcomes of very low birth weight infants (VLBWI) born preterm after maternal preeclampsia and HELLP syndrome within the German Neonatal Network.

**Methods:** The German Neonatal Network is a large population-based cohort study enrolling VLBWI since 2009. Two thousand six hundred and fifty two infants below 32 weeks of gestation born after maternal preeclampsia or HELLP syndrome and 13,383 infants born prematurely for other causes between 2009 and 2018 were included in our analysis. Descriptive statistics and multinomial regression models including preeclampsia and HELLP syndrome were performed for short-term outcome measures such as intracerebral hemorrhage, necrotizing enterocolitis requiring surgery, bronchopulmonary dysplasia, retinopathy of prematurity, periventricular leukomalacia, persistent ductus arteriosus requiring surgery, blood culture positive sepsis and death.

**Results:** After adjustment for confounding variables, preterm birth due to preeclampsia or HELLP syndrome was associated with a reduced risk for intracerebral hemorrhage (OR 0.73, 95% CI 0.60–0.89), necrotizing enterocolitis requiring surgery (OR 0.35 95% CI 0.15–0.82), periventricular leukomalacia (OR 0.61 95% CI 0.40–0.92), and death (OR 0.72 95% CI 0.55–0.96) as compared to other causes of preterm birth.

**Conclusions:** The indication for preterm birth has an impact on neonatal outcome in preterm infants born below 32 weeks. This notion should be included when counseling the families.

## Introduction

Globally, about 11% of all infants are born preterm, but the rates significantly vary between different countries and continents (1). In Germany, 8.6% of all children were born preterm in 2017 (2). Very low birth weight infants (birth weight <1,500 g, VLBWI) have the most critical outcome which is influenced by several known risk factors like gestational age, birth weight, antenatal exposure to corticosteroids, gender, single/multiple gestation, place of birth, and mode of delivery (3–5).

Hypertensive disorders are one of the world's leading causes of maternal and perinatal mortality (6). Preeclampsia complicates 2–8% of all pregnancies globally (7) and is characterized by the new onset combination of hypertension after 20 weeks of gestation with proteinuria or another organ dysfunction like thrombocytopenia, renal insufficiency, impaired liver function, pulmonary edema or neurological impairment. Hemolysis, elevated liver enzymes and low platelet count (HELLP) syndrome is a severe form of preeclampsia but can also present without hypertension or proteinuria in up to 15% of the patients (8). The pathophysiology of preeclampsia and HELLP syndrome is not completely understood. Most likely, a disturbed implantation of the placenta is leading to uteroplacental ischemia which causes the release of vasoactive substances from the placenta into the maternal circulation, resulting in an endothelial dysfunction (9, 10). As preeclampsia and HELLP syndrome can only be resolved by removal of the placenta, preterm birth is more common in this group (11). Potential complications for the fetus due to the placental dysfunction include intrauterine growth restriction (IUGR), oligohydramnios, and placental abruption (6).

An interdisciplinary perinatal collaboration is crucial to discuss all the risk factors of threatening preterm birth in order to guide counseling and medical decision-making for an optimal timing of delivery. There are many studies showing that indications for delivery like preterm premature rupture of membranes (PPROM) with chorioamnionitis (12) or IUGR (13) have an impact on neonatal outcome. The objective of this study was to determine the impact of preeclampsia and HELLP syndrome on infant survival and morbidity in a large cohort of VLBWI enrolled in the German Neonatal Network (GNN).

## Materials and Methods

### VLBWI Cohort and Data Collection

In our analysis we included data of VLBWI who were born in 62 GNN centers between January 1st, 2009, and December 31st, 2018. Infants with lethal abnormalities were excluded. After written informed consent was obtained from the parents or legal guardians, data was collected prospectively by neonatologists or trained study personnel. A clinical data set including pre-, peri- and post-natal treatment and outcome data was recorded by according data sheets. The cause of preterm birth was determined by the attending obstetrician, multiple causes were possible. All case record forms were sent to the study center at the University of Luebeck. Data quality was evaluated by annual on-site monitoring by a study nurse or a pediatrician experienced in neonatology.

For statistical analysis, we included VLBWI with at least one known reason for preterm birth. Recorded indications for delivery were clinical chorioamnionitis, preterm labor, pathological cardiotocography, IUGR, preeclampsia and HELLP syndrome, abruption of placenta, PPROM as well as “other reasons.” In this analysis, infants born due to preeclampsia or HELLP syndrome were analyzed against all other infants.

### Statistical Analyses

Descriptive statistics using percentages for peri- and post-natal parameters and corresponding indications for preterm birth were carried out. For categorical variables Pearson's-Chi-square test and for continuous variables Mann-Whitney-*U-*test were used for calculating statistical significance. The type I error level was set to 0.05. To test associations between cause of preterm birth (preeclampsia and HELLP syndrome) and different outcome variables we performed a multinomial regression model to calculate odds ratios (OR) and corresponding 95% confidence intervals (CI). Confounding variables included: gestational age, application of antenatal steroids, mode of delivery, gender, birth weight, multiple birth and IUGR. The following outcome variables were tested: intracerebral hemorrhage (ICH), necrotizing enterocolitis (NEC) requiring surgery, bronchopulmonary dysplasia (BPD), retinopathy of prematurity (ROP), periventricular leukomalacia (PVL), persistent ductus arteriosus (PDA) requiring surgery, blood culture positive sepsis and death. Missing data were not imputed. All statistical analyses were performed with SPSS 25.0 software (IBM SPSS Statistics for Windows, Version 25.0. Munich, Germany).

### Definitions

The analyzed group “preeclampsia and HELLP syndrome” comprises all infants who were delivered because of maternal HELLP syndrome, preeclampsia or eclampsia as documented by the attending obstetrician. IUGR is defined as intrauterine growth <10th percentile for gestational age in combination with pathologic arterial and venous Doppler ultrasound examination in pregnancy. The diagnosis was recorded by the attending neonatologist as documented in the maternal medical record by the obstetrician. The term is used distinct from small for gestational age (SGA), which is defined as birth weight <10th percentile according to gestational age (14). IUGR fetuses do not reach their genetically determined growth potential as a consequence of the placental dysfunction and have a higher morbidity and mortality, whereas SGA fetuses include the large group of genetically small fetuses without pathology (15). Clinical chorioamnionitis is a syndrome of the mother, which is diagnosed if one or more of the following signs and symptoms are present: maternal fever (intrapartum temperature >37.8°C), maternal tachycardia (>120 beats/min) or fetal tachycardia (>160–180 beats/min), purulent or foul-smelling amniotic fluid or vaginal discharge, uterine tenderness, maternal leukocytosis (total blood leukocyte count >15,000–18,000 cells/μL) (16).

BPD is diagnosed when needing supplemental oxygen or ventilation support at 36 weeks of post-menstrual age (17). Clinical sepsis was defined as condition when neonatologists decided to treat the infant with antibiotics and continue for at least 5 days due to the following reasons: ≥ 2 clinical signs of systemic inflammatory response: temperature > 38°C or <36.5°C, tachycardia > 200/min, new onset or increased frequency of bradycardias or apnoea, hyperglycaemia > 140 mg/dl, base excess < −10 mval/l, changed skin color, increased oxygen need; and 1 laboratory sign: C-reactive protein > 10 mg/L, platelet count <100/nl, immature/total neutrophil ratio > 0.2, white blood cell count <5/nl (NeoKISS) (18, 19). Clinical diagnosis of early-onset sepsis (EOS) is defined as signs of sepsis within the first 72 h after birth with or without proof of a causative agent in blood culture, clinical diagnosis of late-onset sepsis (LOS) is defined as signs of sepsis after the first 72 h after birth with or without proof of a causative agent. Death is defined as death occurring after admission to the NICU within the primary stay in hospital. Intracerebral hemorrhage grades I-IV are diagnosed according to the ultrasound criteria of Papile in line with a standardized protocol derived from the DEGUM (German Society for Ultrasound in Medicine) (20). NEC requiring surgery is defined as clinical NEC classified as Bell Stage II or Bell Stage III with the need for laparotomy with or without resection of necrotic gut, and the macroscopic diagnosis of NEC. PVL is defined as white-matter brain injury, characterized by cystic degeneration of white matter near the lateral ventricles as diagnosed by ultrasound imaging which was applied in all participating centres. ROP requiring surgery is defined as ROP stage with need of operative treatment.

## Results

### Study Population and Perinatal Characteristics

**Figure 1:**
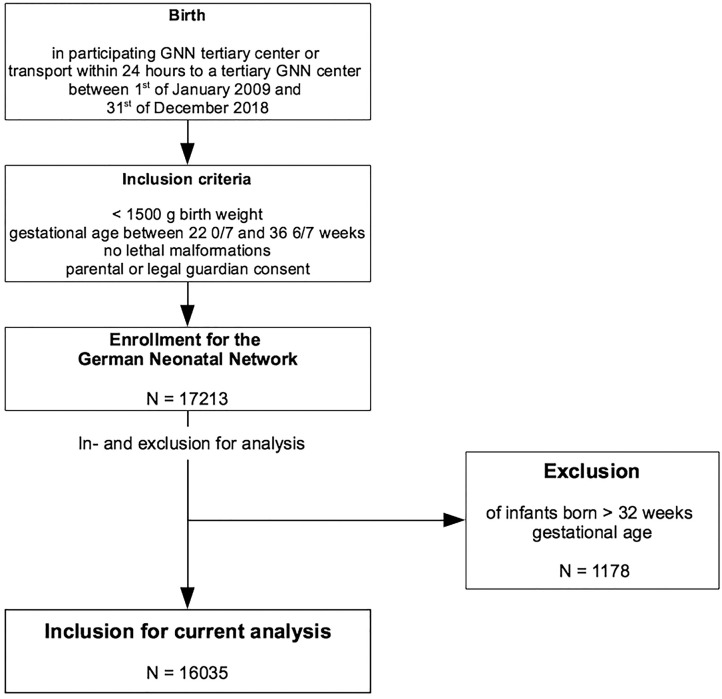
Flowchart of enrolment and inclusion criteria of the study population.

Between 2009 and 2018, 17,213 VLBWI below 37 weeks of gestation were enrolled in the GNN. After exclusion of VLBWI born > 32 weeks, datasets of 16,035 VLBWI were available for analysis. Of these infants, 2,652 were born after maternal preeclampsia or HELLP syndrome (see Figure 1).

Table 1 shows the baseline characteristics of our cohort. Infants born due to preeclampsia and HELLP syndrome had a higher mean gestational age compared to infants with other indications for preterm birth [29.0 (±2.3) vs. 28.1 (±2.5) weeks of gestation]. In the preeclampsia and HELLP group, there were significantly more female infants (53.8 vs. 47.2%) and less vaginal deliveries (0.8 vs. 10.7%).

**Table 1:** Cohort characteristics according to indication for preterm birth.

	**Preeclampsia/HELLP**		**All other indications**		***p*-value**
	***n*** ***=*** **2,652**		***n*** ***=*** **13,383**		
Gestational age at birth, mean weeks (SD)	29.0 (2.3)		28.1 (2.5)		<0.001
Birth weight, mean grams (SD)	1,001 (312)		1,028 (301)		0.001
Female gender, n (%)	1,427 (53.8)	51.9–55.7	6,319 (47.2)	46.4–48.1	<0.001
Multiple gestation, n (%)	365 (13.8)	12.5–15.1	5,261 (39.3)	38.5–40.1	<0.001
Antenatal steroids, n (%)	2,457 (92.9)	91.9–93.8	12,191 (91.2)	90.7–91.7	0.005
**Mode of delivery**					
Vaginal delivery, n (%)	20 (0.8)	0.5–1.1	1,431 (10.7)	10.2–11.3	
Elective cesarean section, n (%)	2,454 (92.9)	91.9–93.8	10,396 (77.9)	77.2–78.6	<0.001*
Emergency cesarean section, n (%)	168 (6.4)	5.5–7.3	1,525 (11.4)	10.9–12.0	

Data are given as mean (SD) or n (%) with 95% confidence interval. Percentages are given as column percentages; *for all modes of delivery.

Table 2 shows additional indications for preterm birth for both groups (preeclampsia and HELLP syndrome vs. all other indications for preterm birth). Pathological cardiotocography (23.6 vs. 20.2%) and IUGR (30.9 vs. 19.5%) were more frequently present in VLBWI delivered after maternal preeclampsia and HELLP syndrome than in VLBWI without preeclampsia and HELLP. This is in line with the fact that IUGR and pathological CTGs may also be a result of a placental dysfunction.

**Table 2:** Indications for preterm birth of the cohort other than preeclampsia and HELLP syndrome.

	**Preeclampsia/HELLP** ***N*** ***=*** **2,652**		**All other indications** ***N*** **=13,383**	
	**n (%)**	**95% CI**	**n (%)**	**95% CI**
HELLP	1,347 (50.9)	49.0–52.8	n.a.	n.a.
Preeclampsia	1,493 (56.3)	54.4–58.2	n.a.	n.a.
Preterm labor	56 (2.1)	1.6–2.7	5,881 (44.1)	43.2–44.9
Clinical chorioamnionitis	51 (1.9)	1.5–2.5	3,565 (26.7)	26.0–27.5
Pathological cardiotocography	623 (23.6)	22.0–25.3	2,694 (20.2)	19.5–20.9
IUGR	817 (30.9)	29.2–32.7	2,602 (19.5)	18.8–20.2
PPROM	5 (0.7)	0.3–1.5	646 (14.4)	13.4–15.4
Placental abruption	74 (2.8)	2.2–3.5	1,135 (8.5)	8.0–9.0
Cervical insufficiency	3 (0.4)	0.1–0.7	315 (7.5)	6.7–8.3
Other reasons	240 (9.0)	8.0–10.2	2,492 (18.6)	18.0–19.3

*Data are given as n (%) with 95% confidence interval. Percentages are given as column percentages*.

### Outcome Characteristics

#### Univariate Analyses

Univariate analyses suggested that infants with maternal preeclampsia and HELLP syndrome had a smaller risk for clinical sepsis (26.4 vs. 29.6%, *p* < 0.001), LOS (10.3 vs. 12.1%, *p* = 0.01), blood culture positive sepsis (10.7 vs. 12.8%, *p* = 0.001), ICH (10.7 vs. 20.2%, *p* < 0.001), PVL (1.4 vs. 3.4%, *p* < 0.001), NEC requiring surgery (1.9 vs. 2.7%, *p* = 0.016), PDA requiring surgery (2.7 vs. 4.2%, *p* < 0.001), and death (2.8 vs. 4.0%, *p* = 0.003) as compared to infants born due to other reasons (Table 3).

**Table 3:** Neonatal outcome parameters by indication for preterm birth.

	**Preeclampsia/HELLP**		**All other indications**		***p*-value**
	***n*** ***=*** **2,652**		***n*** ***=*** **13,383**		
	**n (%)**	**95% CI**	**n (%)**	**95% CI**	
Inotropes first 24 h	120 (6.4)	5.3–7.5	1,025 (10.6)	10.0–11.2	<0.001
Clinical sepsis	698 (26.4)	24.7–28.1	3,963 (29.6)	28.9–30.4	0.001
Early-onset sepsis	261 (11.6)	10.3–12.9	1,476 (12.9)	12.3–13.6	0.072
Late-onset sepsis	273 (10.3)	9.2–11.5	1,616 (12.1)	11.5–12.6	0.010
Blood culture proven sepsis	283 (10.7)	9.6–11.9	1,713 (12.8)	12.3–13.4	0.002
BPD	368 (13.9)	12.7–15.3	2,030 (15.3)	14.7–15.9	0.087
Oxygen need at discharge	199 (7.5)	6.6–8.6	1,138 (8.5)	8.1–9.0	0.089
ICH	283 (10.7)	9.5–11.9	2,693 (20.2)	19.5–20.8	<0.001
PVL	38 (1.4)	1.0–1.9	456 (3.4)	3.1–3.7	<0.001
NEC requiring surgery	51 (1.9)	1.5–2.5	366 (2.7)	2.5–3.0	0.016
PDA requiring surgery	72 (2.7)	2.1–3.4	561 (4.2)	3.9–4.5	<0.001
Death	75 (2.8)	2.2–3.5	539 (4.0)	3.7–4.4	0.003

*Data are given as n (%) with 95% confidence interval. Percentages are given as column percentages*.

VLBWI born after preeclampsia and HELLP syndrome with IUGR showed increased frequencies for complications compared to infants born without IUGR (Table 4), in particular increased rates of clinical sepsis (33 vs. 23.4%, *p* < 0.001), blood culture proven sepsis (13.0 vs. 9.7%, *p* = 0.012), BPD (19.1 vs. 11.6%, *p* < 0.001), NEC requiring surgery (3.3 vs. 1.3%, *p* = 0.001), and death (4.4 vs. 2.1, *p* = 0.001).

**Table 4:** Outcome of infants born after preeclampsia or HELLP syndrome with or without intrauterine growth restriction.

	**IUGR** ***N*** ***=*** **817**		**No IUGR** ***N*** ***=*** **1,823**		***p*-value**
	**n (%)**	**95% CI**	**n (%)**	**95% CI**	
Clinical sepsis	269 (33.0)	29.8–36.2	426 (23.4)	21.5.−25.4	<0.001
Early-onset sepsis	99 (14.2)	11.8–17.0	159 (10.3)	8.8–11.8	0.006
Late-onset sepsis	106 (13.0)	10.8–15.4	167 (9.2)	7.9–10.6	0.003
Blood culture positive sepsis	106 (13.0)	10.8–15.4	177 (9.7)	8.4–11.2	0.012
BPD	155 (19.1)	16.5–21.9	211 (11.6)	10.2–13.2	<0.001
ICH	89 (10.9)	8.9–13.2	193 (10.6)	9.2–12.1	0.813
PVL	13 (1.6)	0.9–2.6	25 (1.4)	0.9–2.0	0.659
NEC requiring surgery	27 (3.3)	2.2–4.7	24 (1.3)	0.9–1.9	0.001
PDA requiring surgery	26 (3.2)	2.1–4.6	46 (2.5)	1.9–3.3	0.335
Death	36 (4.4)	3.2–6.0	38 (2.1)	1.5–2.8	0.001

*Data are given as numbers (%) with 95% confidence interval. Percentages are given as column percentages*.

When evaluating all IUGR infants within the cohort (*n* = 3,428 in total), the neonates who were additionally exposed to maternal preeclampsia and HELLP syndrome had higher frequencies for clinical sepsis (33.0 vs. 27.1%, *p* = 0.001), BPD (19.1 vs. 15.3%, *p* = 0.011), and NEC requiring surgery (3.3 vs. 2.0%, *p* = 0.025) compared to VLBWI with IUGR but without preeclampsia and HELLP syndrome (Table 5).

**Table 5:** Univariate analysis of outcome parameters of all infants born after IUGR with or without preeclampsia and HELLP syndrome.

	**Preeclampsia/HELLP** ***N*** ***=*** **826**		**No preeclampsia/HELLP** ***N*** ***=*** **2,602**		***p*-value**
	**n (%)**	**95% CI**	**n (%)**	**95% CI**	
Clinical sepsis	269 (33.0)	29.8–36.2	705 (27.1)	25.4–28.8	0.001
Early-onset sepsis	99 (14.2)	11.8–17.0	236 (10.4)	9.5–12.1	0.012
Late-onset sepsis	106 (13.0)	10.8–15.4	275 (10.6)	9.4–11.8	0.055
Blood culture positive sepsis	106 (13.0)	10.8–15.4	276 (10.6)	9.5–11.8	0.061
BPD	155 (19.1)	16.5–21.9	396 (15.3)	14.0–16.7	0.011
ICH	89 (10.9)	8.9–13.2	302 (11.6)	10.4–12.9	0.579
PVL	13 (1.6)	0.9–2.6	64 (2.5)	1.9–3.1	0.145
NEC requiring surgery	27 (3.3)	2.2–4.7	51 (2.0)	1.5–2.6	0.025
PDA requiring surgery	26 (3.2)	2.1–4.6	77 (3.0)	2.4–3.7	0.747
Death	36 (4.4)	3.2–6.0	99 (3.8)	3.1–4.6	0.441

*Data are given as n (%) with 95% confidence interval*.

#### Multinomial Regression

In our final analysis we adjusted for confounding factors, which are known to influence the outcome of VLBWI, such as gestational age, antenatal administration of steroids, mode of delivery, gender, birth weight, multiple birth, IUGR as well as preeclampsia, and HELLP syndrome. We found that preeclampsia and HELLP syndrome were associated with a risk reduction for ICH (OR 0.73 95% CI 0.60–0.89, *p* = 0.002), NEC requiring surgery (OR 0.35 95% CI 0.15–0.82, *p* = 0.016), PVL (OR 0.61 95% CI 0.40–0.92, *p* = 0.017), and neonatal death (OR 0.72 95% CI 0.55–0.96, *p* = 0.023, Table 6).

**Table 6:** Multinomial regression for different outcome variables controlled for gestational age, antenatal administration of steroids, mode of delivery, gender, birth weight, multiple birth, IUGR and preeclampsia, and HELLP syndrome.

	**Intracerebral** **hemorrhage**		**Necrotizing** **enterocolitis**^**§**^		**Bronchopulmonary** **dysplasia**		**Retinopathy of** **prematurity**^**§**^		**Periventricular** **leukomalacia**		**Persistent ductus** **arteriosus**^**§**^		**Blood culture** **positive sepsis**		**Death**	
	**OR (95%CI)**	***p*-value**	**OR (95%CI)**	***p*-value**	**OR (95%CI)**	***p-*value**	**OR (95%CI)**	***p*-value**	**OR (95%CI)**	***p*-value**	**OR (95%CI)**	***p*-value**	**OR (95%CI)**	***p*-value**	**OR (95%CI)**	***p*-value**
**Gestational age**	**0.70 (0.67–0.74)**	**<** **0.001**	**0.73 (0.62–0.86)**	**<0.001**	**0.80 (0.76–0.85)**	**<0.001**	**0.60 (0.52–0.69)**	**<0.001**	**0.68 (0.62–0.75)**	**<0.001**	**0.59 (0.54–0.65)**	**<0.001**	**0.70 (0.67–0.73)**	**<0.001**	**0.70 (0.65–0.75)**	**<0.001**
**Antenatal steroids**	**0.50 (0.42–0.61)**	**<0.001**	0.70 (0.35–1.40)	0.307	0.83 (0.65–1.06)	0.145	**0.49 (0.31–0.76)**	**0.002**	**0.45 (0.32–0.62)**	**<0.001**	**0.47 (0.35–0.64)**	**<0.001**	**0.67 (0.55–0.80)**	**<0.001**	**0.41 (0.31–0.54)**	**<0.001**
**Mode of delivery**	1.00 (0.88–1.13)	0.930	0.93 (0.61–1.42)	0.731	1.14 (0.99–1.31)	0.065	1.05 (0.79–1.41)	0.730	1.17 (0.93–1.47)	0.193	1.20 (0.98–1.47)	0.079	1.04 (0.93–1.17)	0.469	1.02 (0.85–1.22)	0.831
**Female Gender**	**0.82 (0.73–0.92)**	**0.001**	**0.51 (0.34–0.77)**	**0.001**	**0.59 (0.52–0.68)**	**<0.001**	**0.56 (0.41–0.76)**	**<0.001**	0.89 (0.71–1.11)	0.295	**0.80 (0.65–0.98)**	**0.033**	**0.76 (0.68–0.85)**	**<0.001**	**0.50 (0.41–0.59)**	**<0.001**
**Birth weight**	**1.00 (1.00–1.00)**	**<0.001**	1.00 (1.00–1.00)	0.086	**1.00 (1.00–1.00)**	**<0.001**	**1.00 (1.00–1.00)**	**<0.001**	1.00 (1.00–1.00)	0.244	**1.00 (1.00–1.00)**	**<0.001**	**1.00 (1.00–1.00)**	**<0.001**	**1.00 (1.00–1.00)**	**<0.001**
**Multiple birth**	1.06 (0.93–1.20)	0.402	1.22 (0.80–1.85)	0.351	0.97 (0.85–1.12)	0.693	0.93 (0.66–1.30)	0.675	1.12 (0.88–1.42)	0.354	1.06 (0.85–1.32)	0.620	1.03 (0.92–1.16)	0.594	**1.57 (1.3–1.89)**	**<0.001**
**IUGR**	0.97 (0.80–1.17)	0.708	1.40 (0.77–2.55)	0.272	0.98 (0.82–1.18)	0.823	1.09 (0.68–1.75)	0.716	1.16 (0.81–1.66)	0.411	1.01 (0.73–1.41)	0.942	1.15 (0.98–1.35)	0.087	1.09 (0.84–1.43)	0.524
**Preeclampsia and HELLP**	**0.73 (0.60–0.89)**	**0.002**	**0.35 (0.15–0.82)**	**0.016**	0.84 (0.70–1.00)	0.052	0.79 (0.50–1.26)	0.316	**0.61 (0.40–0.92)**	**0.017**	0.95 (0.69–1.30)	0.735	0.96 (0.82–1.12)	0.583	**0.72 (0.55–0.96)**	**0.023**

Reference category for reason of birth was “all other” reasons, as for example clinical chorioamnionitis, preterm labor, pathological cardiotocography, or abruption of placenta.

§ *requiring surgery. For better reading, significant values are given in bold*.

## Discussion

We present observational data of the large-scale multicenter GNN cohort evaluating the effects of maternal preeclampsia and HELLP syndrome on morbidity and mortality of VLBWI born before 32 weeks of gestation. Our data suggest that VLBWI born after maternal preeclampsia and HELLP syndrome have a lower risk for ICH, NEC requiring surgery, PVL, and death as compared to infants born due to other reasons. These results remaine significant after controlling for known risk factors like gestational age, antenatal administration of steroids, mode of delivery, gender, birth weight, multiple birth, and IUGR in a multinomial regression model.

The major strengths of our population-based data analysis are the large cohort size in a multicenter setting within Germany (*n* = 2,652 cases of preeclampsia or HELLP syndrome) and the accurate phenotypic characterization of the infants. This is accomplished by regular monitoring of data quality within the GNN. There also are some limitations which need to be discussed. One limitation is the fact that it was common in our cohort to have more than one prenatal cause for preterm birth per infant-mother pair. For example, 56 of 2,652 VLBWI (2.1%) were exposed to preeclampsia and HELLP syndrome in combination with preterm labor (see Table 2). This could state a source of confounding when looking at the possible effects of a reason for preterm birth on neonatal outcome. Only a prospective design which differentiates between the main causes of preterm birth and secondary maternal or fetal morbidities could overcome these limitations. A prospective multicenter study by Garite et al. (21) with 1,089 infants tried to solve this problem by additionally including the maternal indication for admission to the hospital in the analysis, but this data was not available for our cohort. In addition, the focus of the GNN is set on the neonatal information. Therefore, there might be some underreporting of maternal morbidities and certain maternal parameters are not recorded. For example, the GNN does not report maternal weight and height. Maternal obesity is associated with preeclampsia and HELLP syndrome (22) and could be an important parameter to influence neonatal outcome. We were not able to control for this variable. We present data on the early neonatal outcomes here. The GNN is also designed to test neonatal outcome at the age of 5–6 years in a subgroup of infants (e.g., cognitive and motoric test, lung function, visual, and hearing tests). The influence of preeclampsia and HELLP syndrome on long-term outcome variables could not be presented in the current analyses due to an insufficient number of data sets. It is important to study the long-term neonatal outcome for this group in the future to see if the early benefits translate to later life.

Recent studies showed an influence of the reason for preterm birth itself on neonatal outcome (12, 13), which is of importance when counseling the parents confronted with a preterm birth risk as well as when aiming for an optimal timing of delivery (21). In the case of preeclampsia and HELLP syndrome previous studies suggested an improved neonatal outcome compared to other reasons for preterm birth. A retrospective cohort study by Wang et al. (23) (*n* = 528, 23–34 weeks) found that preeclampsia was associated with an improved neonatal outcome compared with fetal and obstetric indications for delivery by using a composite neonatal outcome consisting of death, cord blood pH <7 or base excess < -12, 5-min Apgar ≤ 3, cardiopulmonary resuscitation during resuscitation, culture-proven sepsis, intraventricular hemorrhage, and necrotizing enterocolitis. We found a lower risk for ICH and PVL in VLBWI born after maternal preeclampsia or HELLP syndrome in our cohort. This is in line with the retrospective cohort study of Morsing et al. (24) (*n* = 1,152, <30 weeks) which found that infants born after maternal preeclampsia showed lower odds of ICH (OR 0.17, 95% CI 0.05–0.57). Furthermore, an analysis from the EPIPAGE cohort noted a lower risk for PVL and ICH for infants born after hypertensive disorders than for infants born after preterm labor or PPROM (25). The authors explained their results with the fact that infants born after maternal hypertensive disorders are less exposed to inflammation. The etiology of adverse neonatal outcome is multifactorial but inflammatory processes are discussed to play a critical role (26). Some authors have reported a higher neonatal infectious morbidity and mortality after PPROM (27), others had contrary results and did not find a negative impact (28, 29). In our cohort, infants born due to other indications included preterm neonates born after spontaneous preterm birth caused by preterm labor (44.1%) and PPROM (14.4%) as well as suspected clinical chorioamnionitis (26.7%, see Table 2). Infants born due to maternal preeclampsia and HELLP syndrome had low rates of preterm labor (2.1%), PPROM (0.7 %) and clinical chorioamnionitis (1.9%, see Table 2) as they were a result of indicated delivery. It is unkown how many of suspected chorioamnionitis cases translated into neonatal inflammation as we did not have histopathological data of the feto-placental unit. We cannot distinguish if inflammation is the reason for the beneficial outcome of the preeclampsia and HELLP group. But if that was the case, one would expect a lower rate of infectious complications like EOS or LOS in the preeclampsia and HELLP group, which we did not find. Further studies are needed to evaluate the impact of maternal clinical chorioamnionitis on neonatal outcome.

Apart from inflammation, the improved outcome of VLBWI with indicated preterm birth for maternal indications has been discussed to be a result of a high rate of cesarean deliveries (30, 31). Typically, women with early-onset preeclampsia do not present with signs of spontaneous preterm birth or infection. 92.9% of VLBWI with preeclampsia and HELLP in our cohort were delivered by cesarean section in contrast to only 77.9% without preeclampsia and HELLP syndrome. However, even after adjustment for mode of delivery in our multinomial regression, the association of preeclampsia and HELLP syndrome with a reduced risk for an adverse neonatal outcome remained statistically significant.

After adjustment for confounders we found no impact of maternal preeclampsia and HELLP syndrome on neonatal BPD risk. There have been descriptions of a relationship between maternal preeclampsia and neonatal BPD as both diseases are associated with signs of impaired angiogenesis, but the results are conflicting (32). Circulating angiogenic factors in the bloodstream of mothers with preeclampsia can cross the placenta, reach the fetus and possibly affect the developing lungs (33). Some studies reported a higher BPD risk in infants born after preeclampsia (34–36), others a lower risk (32). Similar to our results, an Australian cohort study (*n* = 1,268) did not show a higher risk for neonatal BPD after preeclampsia (37).

In analogy to BPD, there have been discussions about an association between maternal preeclampsia and the neonatal NEC risk. We found a significantly lower risk for NEC requiring surgery in the preeclampsia and HELLP group. Some studies have reported a higher risk for NEC in preeclampsia cohorts (34), but this was often found in the subgroups of IUGR infants of preeclamptic mothers (38, 39). A large case-control study (*n* = 720 cases of NEC) from Sweden reported similar results of a lower NEC risk for infants born after preeclampsia (40) but the underlying mechanisms remain unclear. Our analysis did not include NEC not requiring surgery, which could also make a difference when comparing our results to other cohorts.

IUGR alone is known to be associated with severe neonatal mortality and morbidity (41, 42). In combination with preeclampsia and HELLP syndrome, growth restricted neonates have a poorer outcome concerning severe morbidity and death (43). Uteroplacental dysfunction is the common pathology leading to IUGR and preeclampsia. Therefore, IUGR is a lot more prevalent in the preeclampsia and HELLP group compared to the control group (30.9 vs. 19.5%) and we had to control for this risk factor in our multinomial regression. Morbidity and mortality of IUGR infants are significantly related to the gestational age at birth. Therefore, the primary goal of obstetrical surveillance and management of preterm growth-restricted fetuses is to delay delivery in order to gain gestational age (44, 45). This is commonly not possible if IUGR is combined with maternal preeclampsia or HELLP syndrome as the time from diagnosis of IUGR to delivery is shorter and the indication for delivery is often made for maternal symptoms. This leads to a poorer neonatal outcome (43). Our univariate analysis shows that VLBWI with IUGR had significantly increased rates of all kinds of sepsis and BPD, NEC requiring surgery and death, which is in concordance with the literature. In the multinomial analysis we controlled for IUGR.

In summary, our study shows that the prenatal diagnosis of preeclampsia and HELLP syndrome leading to preterm birth has an impact on neonatal outcome. In our cohort, VLBWI born after preeclampsia and HELLP syndrome have a lower risk for ICH, PVL, NEC, and death. This information can be used in interdisciplinary discussions in order to guide counseling and medical decision-making leading toward an optimal timing for preterm delivery. Further prospective studies which define a main indication for delivery are necessary to study the impact of the indication of delivery on neonatal outcome.

## Data Availability Statement

Due to the inclusion of genetic data, the datasets analysed in this study are not publicly available. However, a reduced dataset based on the analysis is available from the corresponding author on reasonable request.

## Ethics Statement

The studies involving human participants were reviewed and approved by the ethics committee for research in human subjects of the University of Luebeck (file number 08-022), Luebeck, Germany, and by the local ethics committees of all participating centers. Written informed consent to participate in this study was provided by the participants' legal guardian.

## Author Contributions

AH and VB: study concept and design. All GNN sites: acquisition of data. AH and TR: statistical analyses. All authors analysis and interpretation of data. VB and AH: drafting of the manuscript. All authors critical revision of the manuscript for important intellectual content. WG: obtained funding and study supervision. All authors contributed to manuscript revision and approved the final version.

## Conflict of Interest

The authors declare that the research was conducted in the absence of any commercial or financial relationships that could be construed as a potential conflict of interest.

## Abbreviations

BPD: bronchopulmonary dysplasia
CI: confidence interval
DEGUM: German Society for Ultrasound in Medicine
EOS: early-onset sepsis
GNN: German Neonatal Network
HELLP: hemolysis, elevated liver enzymes, low platelet count
ICH: intracerebral hemorrhage
IUGR: intrauterine growth restriction
LOS: late-onset sepsis
NEC: necrotizing enterocolitis
OR: odds ratio
PDA: persistent ductus arteriosus Botalli
PVL: periventricular leukomalacia
ROP: retinopathy of prematurity
SGA: small for gestational age
VLBWI: very low birth weight infant.
